# Establishing Pandemic Influenza Severity Assessment (PISA) parameters and thresholds for Canada’s FluWatch program

**DOI:** 10.14745/ccdr.v49i1112a04

**Published:** 2023-11-01

**Authors:** FluWatch Team

**Affiliations:** 1 Public Health Agency of Canada, Ottawa, ON

**Keywords:** seasonal influenza, severity, FluWatch, PISA, thresholds

## Abstract

**Background:**

The World Health Organization (WHO) developed a structured framework to enable countries to rapidly assess the severity of an influenza pandemic. This framework, the Pandemic Influenza Severity Assessment (PISA), is intended to be performed weekly during seasonal epidemics so that assessing influenza severity during a pandemic can be done with greater ease and efficiency.

**Objective:**

Using influenza surveillance indicators within Canada’s FluWatch program from seasons 2014–2015 to 2018–2019, national PISA thresholds were developed and assessed against seasonal data for seasons 2019–2020 to June of 2022–2023.

**Outcomes:**

Canada developed thresholds for each required indicator (transmissibility, seriousness of disease and impact) for multiple WHO-recommended parameters. The thresholds were assessed against four seasons, and it was determined that there was a good agreement between the PISA assessments and the characterization of the season by FluWatch epidemiologists.

**Conclusion:**

With confidence in the validity of the PISA thresholds, the FluWatch program will begin to share PISA assessments weekly through the FluWatch report in the 2023–2024 seasons to help characterize influenza activity in Canada and inform responses to the seasonal influenza epidemic.

## Introduction

One of the major gaps of the 2009 H1N1 pandemic was the ability of Member States of the World Health Organization (WHO) to rapidly assess the severity of the pandemic. To address this gap and to better prepare for future influenza pandemics, the WHO developed a structured framework to assess influenza severity, known as the Pandemic Influenza Severity Assessment (PISA) ((1)).

From 2014 to 2016, Canada participated in a WHO pilot that assessed the interim framework for PISA, and this framework was further refined based on the results from this pilot. In 2017, an official PISA guidance document was published by the WHO for Member States to implement PISA in their respective regions. Assessments are meant to be performed weekly during seasonal epidemics, and outputs are shared with the WHO and used in routine seasonal influenza situational assessments and reports. The goal is for a country to use PISA during seasonal epidemics so that assessing severity during a pandemic can be done with greater ease and efficiency.

In this article, the implementation of PISA into Canada’s national influenza surveillance program, FluWatch, is summarized. In addition to implementing PISA for influenza indicators, PISA was also applied to respiratory syncytial virus (RSV) to determine whether PISA thresholds could be developed for viruses other than influenza.

## Methods

### Data sources

FluWatch is a long-standing national surveillance system that monitors the spread of influenza and influenza-like illness (ILI) in Canada. FluWatch is a composite surveillance system consisting of virological surveillance, influenza and ILI activity level surveillance, syndromic surveillance, outbreak surveillance, severe outcome surveillance, and vaccine monitoring ((2)). All the data that was considered for PISA originated from the FluWatch surveillance system.

Indicators and parameters for assessment are outlined in the PISA guidance document (1) and in subsequent refinements that were shared among the Member States at working group meetings ((3)). A list of each recommended parameter and associated indicator is found in **Table 1**. The PISA framework defines influenza severity through the use of three indicators: Transmission, Seriousness, and Impact. Each indicator consists of multiple parameters that countries can use. It is not required for each country to have every parameter listed by the WHO and it is up to each country to decide which parameters to monitor:

**Table 1:** WHO PISA parameters, FluWatch indicators and data sources used, Canada, season 2014–2015 to 2018–2019

WHO recommended parameter	FluWatch indicator	FluWatch data source
**Transmissibility**		
Weekly ILI or MAARI cases as a proportion of total visits or incidence rate	% visits for ILI	Sentinel Primary Care Provider ILI Surveillance Program
Composite (product) of weekly ILI or MAARI rates and weekly percentage positivity for influenza	N/A	Data not available (ILI rates and percentage positivity data from FluWatch indicators do not come from the same sites)
Percentage positivity from specific syndromic presentations (e.g., ILI, ARI, MAARI)	% positive for influenza % positive for RSV	Respiratory Virus Detection Surveillance System (RVDSS)
Number of influenza or respiratory outbreaks reported in care facilities housing elderly or other susceptible groups	Number of laboratory-confirmed influenza outbreaks	Outbreak surveillance
Other healthcare system usage for mild respiratory illness	N/A	Data not available
Data from participatory surveillance	% FluWatchers participants reporting cough and fever	FluWatchers^a^
**Seriousness**		
Cumulative death: hospitalization ratio	Number of in-hospital influenza deaths (all cases and by ages 0–19, 20–64 and 65+) Number of influenza hospitalizations (all cases and by ages 0–19, 20–64 and 65+)	Provincial and Territorial Severe Outcome Surveillance (PTSOS)
Cumulative ICU: hospitalization ratio	Number of influenza ICU admissions (all cases and by ages 0–19, 20–64 and 65+) Number of influenza hospitalizations (all cases and by ages 0–19, 20–64 and 65+)	PTSOS
SARI:ILI or SARI:ARI ratios	N/A	Data not available
**Impact – Morbidity and Mortality**		
Weekly number of hospital or ICU admissions for influenza/SARI/respiratory illness, or rate per unit population	Number of influenza hospitalizations (all cases and by ages 0–19, 20–64 and 65+)^b^ Number of influenza ICU admissions	PTSOS
SARI proportion or influenza-confirmed SARI proportion of all hospital or ICU admissions	N/A	Data not available
Number of patients currently in hospital or ICU with influenza/SARI/respiratory illness, or rate per unit population	N/A	Data not available
Composite (product) of weekly SARI rate and weekly percentage positivity rates of SARI cases for influenza	N/A	Data not available
Weekly excess pneumonia and influenza or all-cause mortality	N/A	Data not available
Number of hospitalizations for influenza/SARI/respiratory illness requiring oxygen support	N/A	Data not available
**Impact – Healthcare Capacity**		
Proportion of all (occupied and available) hospital or ICU beds currently occupied for influenza/SARI/respiratory illness or all causes	N/A	Data not available
Proportion of beds with oxygen support occupied for influenza/SARI/respiratory illness or all causes	N/A	Data not available
Healthcare workforce absenteeism	N/A	Data not available
Saturation of primary healthcare capacity	N/A	Data not available

Abbreviations: ARI, acute respiratory infection; ICU, intensive care unit; ILI, influenza-like illness; MAARI, medically attended acute respiratory infection; N/A, not applicable; PISA, Pandemic Influenza Severity Assessment; PTSOS, Provincial and Territorial Severe Outcome Surveillance; RVDSS, Respiratory Virus Detection Surveillance System; RSV, respiratory syncytial virus; SARI, Severe Acute Respiratory Infection; WHO, World Health Organization

^a^ FluWatchers data are excluded from the analysis, as data is only available for seasons 2016–2017 onwards (3 seasons)

^b^ Rate per unit population can be calculated; however, to keep thresholds consistent with ICU admissions (where rates cannot be calculated), weekly number of hospitalizations will be used

Transmissibility: how many people in a population get sick from influenza on a weekly basis

Seriousness: how severely sick individual people get when infected with the influenza virus

Impact: how the influenza epidemic or pandemic affects the healthcare system and society. As of 2023, Impact is split into two indicators: morbidity and mortality, and impact on healthcare capacity

It is recommended that historical data considered for PISA captures at least five seasons and, when possible, thresholds by age are developed.

To develop the thresholds, data for the seasons 2014–2015 to 2018–2019 from the identified FluWatch surveillance indicators in Table 1 were used. The calculated thresholds were assessed against four seasons: 2019–2020, 2020–2021, 2021–2022, and 2022–2023 (to June). In the 2022–2023 season, the epidemic started and ended early ((4)). Any activity occurring from June to the end of the surveillance season (August 26, 2023) would not have affected the season assessment.

Two methods were used to determine the thresholds: Moving Epidemic Method (MEM) and the WHO method. The MEM method was developed by Vega *et al.* and is recommended by the WHO for Transmissibility and Impact parameters ((1,5)). The WHO has developed an online MEM tool (6). The FluWatch program used the online MEM tool to calculate the thresholds.

The WHO method is recommended for calculating thresholds for Seriousness ((1)). To determine the thresholds for moderate, high, and extraordinary Seriousness, the mean, mean plus 1 standard deviation, and mean plus 3 standard deviations of end-of-season values, respectively, were used. Any value below the mean would be considered low Seriousness. The Seriousness and Impact thresholds were developed using Excel 365 (Microsoft Corp., Redmond, United States).

## Results

### Transmissibility

**Figure 1:**
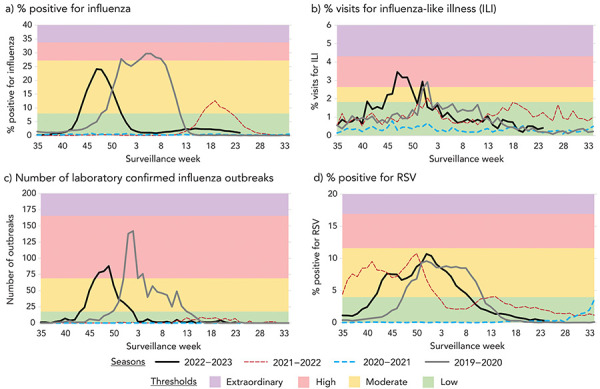
Influenza Transmissibility parameters and thresholds by FluWatch indicator^a^, assessed against data for season 2019–2020 to June 2022–2023 season, Canada Abbreviations: ILI, influenza-like illness; RSV, respiratory syncytial virus ^a^ Figure is divided in the following quadrants: a) percent positive for influenza; b) percent visits for influenza-like illness (ILI); c) number of laboratory confirmed influenza outbreaks; d) percent positive for RSV

**Percentage of tests positive for influenza:** The thresholds for moderate, high and extraordinary were determined to be 8.0, 27.2 and 34.0 (**Figure 1**, a). Any value below 8.0 was considered low. Two seasons (2021–2022 and 2022–2023) peaked at moderate Transmissibility, one season peaked at high Transmissibility (2019–2020) and one season (2020–2021) remained at low Transmissibility for the whole season.

**Percentage of visits for ILI:** The thresholds for moderate, high and extraordinary percentage of visits for ILI were determined to be 1.8, 2.6 and 4.3 (Figure 1, b). Any value below 1.8 was considered low. Two seasons (2019–2020 and 2022–2023) peaked at high Transmissibility, one season peaked at moderate Transmissibility (2021–2022), and one season (2020–2021) remained at low Transmissibility for the whole season.

**Number of laboratory-confirmed influenza outbreaks:** The thresholds for moderate, high and extraordinary were determined to be 18, 69 and 166 (Figure 1, c). Any value below 18 was considered low. Two seasons (2019–2020 and 2022–2023) peaked at high Transmissibility and the other two seasons remained at low Transmissibility for the whole season.

**Percentage of tests positive for RSV:** The thresholds for moderate, high and extraordinary were determined to be 4.0, 11.6 and 16.9 (Figure 1, d). Any value below 4.0 was considered low. Three seasons (2019–2020, 2021–2022 and 2022–2023) peaked at moderate Transmissibility and one season (2020–2021) remained at low Transmissibility for the whole season.

### Seriousness

Mid-season (week 8) and year end (week 34) values are recommended for measuring Seriousness indicators. Mid-season values were the same as the year-end values for each included season. Thresholds for low, moderate, high and extraordinary are outlined in **Table 2** and **Table 3**.

**Table 2:** Cumulative ICU in hospitalization ratio thresholds (Seriousness indicator), by age groups, seasons 2019–2020 to 2022–2023, Canada

Season and age group (years)	Threshold level (ratio range)			
**All ages**	**Low** **(0–0.11)**	**Moderate** **(0.12–0.19)**	**High** **(0.20–0.33)**	**Extraordinary** **(0.34+)**
2022–2023	X	-	-	-
2021–2022	X	-	-	-
2020–2021	X	-	-	-
2019–2020	-	X	-	-
**0–19**	**Low** **(0–0.09)**	**Moderate** **(0.10–0.16)**	**High** **(0.17–0.29)**	**Extraordinary** **(0.30+)**
2022–2023	X	-	-	-
2021–2022	X	-	-	-
2020–2021	X	-	-	-
2019–2020	-	X	-	-
**20–64**	**Low** **(0–0.18)**	**Moderate** **(0.19–0.31)**	**High** **(0.32–0.57)**	**Extraordinary** **(0.58+)**
2022–2023	X	-	-	-
2021–2022	X	-	-	-
2020–2021	X	-	-	-
2019–2020	X	-	-	-
**65+**	**Low** **(0–0.08)**	**Moderate** **(0.09–0.12)**	**High** **(0.13–0.21)**	**Extraordinary** **(0.22+)**
2022–2023	X	-	-	-
2021–2022	X	-	-	-
2020–2021	X	-	-	-
2019–2020	-	X	-	-

Abbreviations: ICU, intensive care unit; -, threshold not reached

**Table 3:** Cumulative death in hospitalization ratio thresholds (Seriousness indicator), by age groups, seasons 2019–2020 to 2022–2023, Canada

Season and age group (years)	Threshold level (ratio range)			
**All ages**	**Low** **(0–0.04)**	**Moderate** **(0.05–0.07)**	**High** **(0.07–0.12)**	**Extraordinary** **(0.13+)**
2022–2023	-	X	-	-
2021–2022	X	-	-	-
2020–2021	X	-	-	-
2019–2020	-	X	-	-
**0–19**	**Low** **(0–0.005)**	**Moderate** **(0.006–0.013)**	**High** **(0.014–0.029)**	**Extraordinary** **(0.030+)**
2022–2023	-	X	-	-
2021–2022	X	-	-	-
2020–2021	X	-	-	-
2019–2020	-	X	-	-
**20–64**	**Low** **(0–0.03)**	**Moderate** **(0.04–0.06)**	**High** **(0.07–0.11)**	**Extraordinary** **(0.12+)**
2022–2023	-	X	-	-
2021–2022	-	X	-	-
2020–2021	X	-	-	-
2019–2020	-	X	-	-
**65+**	**Low** **(0–0.06)**	**Moderate** **(0.07–0.10)**	**High** **(0.11–0.17)**	**Extraordinary** **(0.18+)**
2022–2023	-	-	X	-
2021–2022	X	-	-	-
2020–2021	X	-	-	-
2019–2020	-	X	-	-

Abbreviation: -, threshold not reached

### Cumulative intensive care unit: Hospitalization ratio

**All ages:** Seasons 2020–2021, 2021–2022 and 2022–2023 were classified as low Seriousness. Season 2019–2020 was classified as moderate Seriousness.

**0–19 years:** Seasons 2020–2021, 2021–2022 and 2022–2023 were classified as low Seriousness. Season 2019–2020 was classified as moderate Seriousness.

**20–64 years:** All seasons were classified as low Seriousness.

**65+ years:** Seasons 2020–2021, 2021–2022 and 2022–2023 were classified as low Seriousness. Season 2019–2020 was classified as moderate Seriousness.

### Cumulative deaths: Hospitalization ratio

**All ages:** Seasons 2020–2021 and 2021–2022 were classified as low Seriousness. Seasons 2019–2020 and 2022–2023 were classified as moderate Seriousness.

**0–19 years:** Seasons 2020–2021 and 2021–2022 were classified as low Seriousness. Seasons 2019–2020 and 2022–2023 were classified as moderate Seriousness.

**20–64 years:** Season 2020–2021 was classified as low Seriousness. Seasons 2019–2020, 2021–2022 and 2022–2023 were classified as moderate Seriousness.

**65+ years:** Seasons 2020–2021 and 2021–2022 were classified as low Seriousness. Season 2019–2020 was classified as moderate Seriousness and 2022–2023 was classified as high Seriousness.

### Impact

The thresholds for Impact were calculated using the WHO method.

### Number of weekly hospitalizations

**Figure 2:**
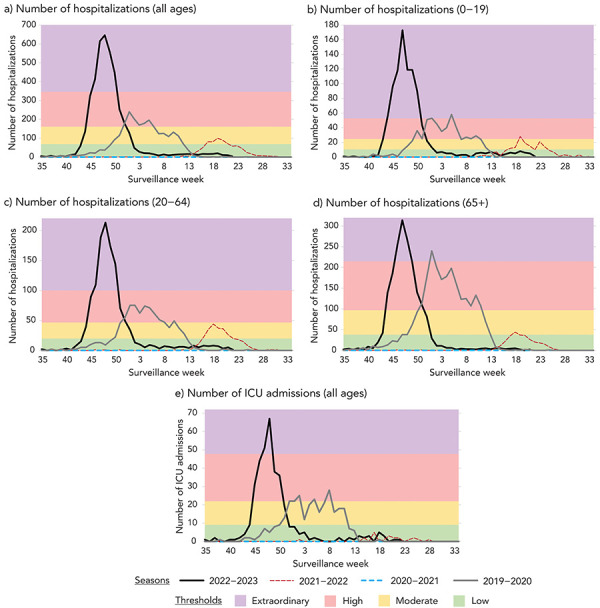
Influenza Impact parameters and thresholds by age group (years, where available) and FluWatch indicator^a^, assessed against data for season 2019–2020 to June 2022–2023 season, Canada Abbreviation: ICU, intensive care unit ^a^ Figure is divided in the following quadrants: a) number of hospitalizations (all ages); b) number of hospitalizations (0–19); c) number of hospitalizations (20–64); d) number of hospitalizations (65+); e) number of ICU admissions (all ages)

**All ages:** The thresholds for moderate, high and extraordinary were determined to be 68, 160 and 346 (**Figure 2**, a). Any value below 68 was considered low. The 2022–2023 season peaked at extraordinary Impact. The 2019–2020 season peaked at high Impact. The 2021–2022 season peaked at moderate Impact. The 2020–2021 season remained at low Impact for the whole season.

**0–19 years:** The thresholds for moderate, high and extraordinary were determined to be 10, 25 and 53 (Figure 2, b). Any value below 10 was considered low. The 2022–2023 and 2019–2020 seasons peaked at extraordinary Impact. The 2021–2022 season peaked at high Impact. The 2020–2021 season remained at low Impact for the whole season.

**20–64 years:** The thresholds for moderate, high and extraordinary were determined to be 68, 160 and 346 (Figure 2, c). Any value below 68 was considered low. The 2022–2023 season peaked at extraordinary Impact. The 2019–2020 season peaked at high Impact. The 2021–2022 season peaked at moderate Impact and the 2020–2021 season remained at low Impact for the whole season.

**65+ years:** The thresholds for moderate, high and extraordinary were determined to be 37, 97 and 214 (Figure 2, d). Any value below 37 was considered low. The 2022–2023 and 2019–2020 seasons peaked at extraordinary Impact. The 2021–2022 season peaked at moderate Impact. The 2020–2021 season remained at low Impact for the whole season.

### Number of weekly intensive care unit admissions

Due to the small weekly numbers, it was determined that measuring intensive care unit (ICU) admissions by age group was not feasible. Instead, ICU admissions were measured as an aggregate of all age groups. The thresholds for moderate, high and extraordinary were determined to be 9, 22 and 48 (Figure 2, e). Any value below 9 was considered low. The 2022–2023 season peaked at extraordinary Impact. The 2019–2020 season peaked at high Impact. The 2019–2020 and 2021–2022 seasons remained at low Impact for the whole season.

## Discussion

The indicators chosen for PISA are reliable, timely and of high quality. With the exception of the healthcare capacity (Impact) indicator, Canada’s FluWatch program has data to support parameters within each indicator, with age-specific parameters for the Seriousness and Impact indicators.

The thresholds resulting from this work allow Canada to assess influenza severity at a national level during both seasonal epidemics and pandemics. PISA is a standardized assessment that is used globally, which allows for country-to-country comparisons and enables Canada to contribute to the WHO’s global severity assessment for influenza.

The Transmissibility indicator has the greatest number of unique parameters (percent positive for influenza, number of laboratory-confirmed outbreaks, percent visits for ILI). The weekly percentage of tests positive for influenza is currently used to determine the start and the end of a seasonal epidemic in Canada; therefore, it is used as the main parameter for Transmissibility. The others will be used as supporting parameters to monitor Transmissibility in different populations (outbreaks—in congregate settings, percent ILI—in the community among those seeking medical care). With additional surveillance seasons available, data from participatory surveillance (FluWatchers) could be added to the Transmissibility indicator as a measure in a population that does not seek medical care.

Two FluWatch indicators (cumulative ICU to hospitalization ratio and cumulative death to hospitalization ratio) were used to assess Seriousness, each stratified by age group (0–19 years, 20–64 years and 65+ years). The availability of age-specific data will allow the FluWatch program to monitor the Seriousness for influenza in different age groups. This indicator requires cumulative data and would be used to assess the season at the midpoint and at the end.

Two FluWatch indicators (number of weekly hospital admissions, number of weekly ICU admissions) were used to assess Impact in the population overall and for three age groups 0–19 years, 20–64 years and 65+ years) for hospitalizations only. The availability of age-specific data in the hospitalization parameter will allow the FluWatch program to monitor impact in different age groups.

The separation of healthcare capacity within the Impact indicator was a recent change in 2023, resulting from the coronavirus disease 2019 (COVID-19) pandemic. Healthcare capacity was an important measure during the COVID-19 pandemic and will likely be an important measure during a future pandemic. Determining a reliable source of data and the accumulation of historical data will be required for the development of this parameter.

The 2019–2020 season was the last pre-pandemic season included in this assessment. The 2019–2020 season peaked at high Transmissibility and Impact, while Seriousness was considered moderate. These assessments are supported by the characterization of the season by the FluWatch program, where the concurrent circulation of all seasonal influenza types and subtypes resulted in higher than average numbers of influenza detections and hospitalizations ((7)). With concurrent circulation of all types and subtypes of influenza, all age groups were affected during that season, which is supported by the moderate levels reported in at least one of the parameters within the Seriousness indicator for each age group.

Due to public health measures implemented for the COVID-19 pandemic, no community circulation of influenza occurred in the 2020–2021 season ((8)). This was evident in the low PISA assessments for the season. Community circulation of influenza returned briefly in the spring of the 2021–2022 season ((9)). This season peaked at moderate Transmissibility while both Impact and Seriousness indicators remained low.

The 2022–2023 season was the first season since the 2019–2020 season where influenza began to return to pre-pandemic circulation patterns. The season started early, with reports of higher than usual influenza-associated hospitalizations, ICU admissions, and deaths ((10)). It peaked at high Transmissibility and extraordinary Impact, while Seriousness was considered both low (for ICU to hospitalization ratio) and moderate (for death to hospitalization ratio). Hospitalization rates were highest among the 65+ and the 0–4 age groups ((10)), wherein high and moderate Seriousness assessments were recorded, respectively.

Transmissibility thresholds within PISA were also developed for RSV. The Transmissibility threshold for RSV will enable the FluWatch program to characterize RSV activity for each season. RSV surveillance has historically been limited to laboratory data; however, there are efforts to expand Canada’s national respiratory surveillance program to include enhanced surveillance indicators for RSV. As RSV surveillance indicators are developed, additional PISA parameters for RSV can also be established. Severe acute respiratory syndrome coronavirus 2 (SARS-CoV-2) could also be another candidate for PISA, as historical endemic/non-pandemic surveillance data accumulates.

The PISA thresholds were developed using pre-pandemic data, which might have an effect on the interpretability and applicability of the thresholds going forward. Upcoming work will include the internal monitoring of the effects of the pandemic on influenza trends and calculated thresholds, as well as determining the appropriateness of including the most recent season’s data (2022–2023) into threshold assessments for future seasons.

## Conclusion

Canada has internally monitored PISA thresholds for the past four seasons. It has been determined that there is good agreement between the PISA assessments and the characterization of the season by FluWatch epidemiologists. The FluWatch program will begin to share PISA assessments in the 2023–2024 FluWatch reports to characterize influenza activity in Canada and to help inform public health responses to seasonal influenza epidemics.
